# Development and validation of a scale on self-regulation in learning (SSRL)

**DOI:** 10.1186/s40064-016-3367-y

**Published:** 2016-09-29

**Authors:** Tolga Erdogan, Nuray Senemoglu

**Affiliations:** 1 Department of Foreign Languages, Land Forces Vocational and Technical NCO School, Cayirhisar, Balikesir Turkey; 2 Division of Curriculum and Instruction, Department of Educational Sciences, Faculty of Education, Beytepe, Ankara, Turkey

**Keywords:** Self-regulation, Motivation, Learning, Scale development, University students, Self-report

## Abstract

Self-regulation is an individual’s influence, orientation, and control over his/her own behaviors. The primary aim of this study was to develop and validate a self-report scale on self-regulation that encompasses both cognitive and motivational factors. The validity and reliability studies of the scale were examined on responses of 872 university students. Exploratory and confirmatory factor analyses confirmed the hypothesized model of self-regulated skills in learning. The scale has 67 items and the factor loadings range from 0.47 to 0.91. The Cronbach’s Alpha was computed 0.91 for the whole scale. Discussions and suggestions parallel to findings are given in the end.

## Background

Zimmerman describes academically successful students as those who approach learning tasks confidently, diligently and are equipped with necessary learning skills. They are also aware of what knowledge or skills they have or not. They are the ones who display a proactive approach towards obtaining information and take giant steps towards mastery of knowledge. Additionally, they find a way to deal with obstacles to learning like bad study conditions, teachers that are confusing, or books that are difficult to understand. Eventually, they perceive learning as a systematic and controllable process and take more responsibility in achieving their objectives (1990: 4–5).

Those students, who are “metacognitively, motivationally, and behaviorally active participants in their own learning process” (Zimmerman 2001: 5; Zimmerman and Schunk 2011), plan the acquisition process, define their objectives, organize information, and continuously monitor and evaluate themselves. With a high level of self-efficacy and strong intrinsic interests towards learning tasks, they choose, design and create learning environments to maximize their own learning (Zimmerman 1990: 4–5; Zimmerman and Martinez-Pons 1988: 284). These behaviors, which are the main focus of this study, are widely known as self-regulated learning skills (SRL).

### Self-regulation/self-regulated learning

Self-regulation is one of the key concepts in Bandura’s *Social Learning Theory* and is described by Senemoğlu as “an individual’s influence, orientation, and control over his/her own behaviors” (2005: 231). Additionally, Zimmerman (2000: 14) formulates self-regulation as “self-generated thoughts, feelings, and actions that are planned and cyclically adapted to the attainment of personal goals”.

From the social cognitive perspective, self-regulation is an interaction of personal, behavioral and environmental processes (Bandura 1986). Zimmerman states that feedback derived from prior performance has an influence over subsequent efforts. These adjustments are inevitable because personal, environmental, and behavioral processes constantly change during the course of learning (Zimmerman 2000: 13–15). As he also depicts; while behavioral self-regulation involves self-observation and strategic adjustment of performance processes like an individual’s learning method, the environmental self-regulation entails the observation and adjustment of environmental conditions or outcomes. Finally, covert self-regulation embraces cognitive and affective states like imagery for remembering or relaxing.

There is a need to identify those self-regulated learning strategies or skills that would help us define individuals as self-regulated learners. In various studies, it is stressed that learners need to believe in the usefulness of such strategies. McCombs (2001) states that SRL is a common function of cognitive and metacognitive strategies together with motivation control and emotion control. In addition to cognitive and metacognitive strategies, there are several other researchers who have highlighted the influence of learners’ beliefs, expectations and their attributions to success and failure on learning (Dweck 1986). Pintrich and De Groot (1990) draw attention to the inadequacy of cognitive and metacognitive strategies without the motivational factors involved in learning. Zimmerman (1990: 11) emphasizes that “self-regulation requires more than cognitive skills; it requires a will or a motivational component as well”. Senemoğlu (2005) highlights the fact that some students may fail even though they make use of appropriate cognitive skills and she points to possible motivational or emotional factors involved. Zimmerman (1990: 6) postulates that self-regulated learning could not be fully understood if learning skills and motivation are treated as independent processes, not interdependent ones. Boekaerts and Cascallar (2006) describe emotion regulation as an important aspect of self-regulation. In her six-component model of SRL, Boekaerts (1996: 102–103) conceptualizes two parallel but strongly interrelated regulatory systems, namely cognitive self-regulation and motivational self-regulation.

In the light of such previous studies and explanations, the framework of a scale that includes possible components of self-regulation would/should be formed under two major components/sections: self-regulated learning strategies and motivational dimensions. The components involved in these two sections and their functions are explained in the following paragraphs.

#### Self-regulated learning skills/strategies

Among various other studies on regulation of learning (Hadwin and Oshige 2011; Sameroff 2010), the authors of the present study decided to form the dimensions of SRL Skills/Strategies by taking basis the models proposed by two groups of researchers, who have formed up their dimensions according to principles of social cognitive theory. In the first self-regulated learning skills model, Zimmerman and Martinez-Pons propose 14 dimensions (Zimmerman and Martinez-Pons 1986: 618; Zimmerman 1989: 337). The second model belongs to Pintrich and De Groot with three broad dimensions. The dimensions in both models are presented in Table 1.

**Table 1:** A comparison of dimensions of the two models of self-regulated learning skills

Zimmerman and Martinez-Pons (1986)	Pintrich and De Groot (1990)
Self-evaluation	*Cognitive learning strategies*
Organizing and transforming	Rehearsal
Goal setting and planning	Elaboration
Seeking information	Organization
Keeping records and self-monitoring	*Metacognitive learning strategies*
Environmental structuring	Planning
Self-consequences	Monitoring
Rehearsing and memorizing	Regulation
Seeking social assistance (peers, teachers, and adults)	*Resource management strategies*
Reviewing records (tests, notes, and textbooks)	Control and management of environment

#### Motivational dimensions

When the motivational dimensions, which are in constant interaction with and are considered sine qua non of self-regulation, are taken into account, the following were decided to be included in the present study: self-efficacy, goal orientations, task value, attributions for failure, and anxiety. The relations of these motivational dimensions to self-regulation and their components are given below.

##### Self-efficacy

Self-efficacy is considered as one of the fundamental motivational factors for self-regulation (Bandura 1986; Schunk 2001; Zimmerman 1989, 1990). The findings of several studies (Pintrich and De Groot 1990) have shown the positive relationship between self-efficacy and self-regulated learning. Students with high self-efficacy were found to be utilizing self-regulated learning skills more than those with low self-efficacy (Pintrich 1999; Wolters 1998).

##### Goal orientations

It is asserted that students may have different goal orientations depending on individual needs and capacities, or situational factors (Meece et al. 1988: 514). While learning-oriented (or mastery-oriented) students try to learn something new and improve their competence, the students with performance goals (social or ego goals) thrive to gain positive judgments about their competence or tend to avoid negative ones. Research has shown that those students with learning goals use self-regulated learning skills more and show more task persistence than those students with performance goals, “who likely choose less challenging tasks where they could demonstrate competence even though they might not learn anything new” (Meece 1994: 29).

##### Task value

The three components of task value (the individual’s perception of the importance of the task, their personal interest in the task, and their perception of the utility value of the task for future goals) (Eccles 1983; in Pintrich 1999: 465) are considered to be positively correlating with the use of self-regulated learning skills (Pintrich 1999: 467; Pintrich and De Groot 1990). Additionally, Schiefele (1991: 311–312) points out that students with high interest and value towards school subjects tend to have “deep level” rather than “surface level” learning, they try to relate material to prior knowledge, and spend much time and effort on learning tasks.

##### Attributions for failure

Licht and Dweck (1984) stress that “helpless” students with performance-oriented goals have different attributions for failure than students with learning goals. Weiner (1979) lists the general attributions for failure as effort, ability, task difficulty, and luck and postulates that learning-oriented students more often see effort as an attribution for failure. Those students who rate those *uncontrollable* factors (luck, ability, and task difficulty) as their reasons for failure or success have no learning tendencies; hence spend less time on task completion and show less persistence. On the contrary, those students who attribute their failures to effort (the *controllable* factor) are willing to take responsibility of their own learning and tend to believe that it is “lack of effort”, not their inability that causes failure (Licht and Dweck 1984; Weiner 1979).

##### Anxiety

Zimmerman (1989: 333) highlights that an affective state like anxiety can impede self-regulated learning and undermine various cognitive and metacognitive learning processes. Pintrich and De Groot (1990) add anxiety as an emotional component to the dimension of student motivation towards academic achievement. Even though the same authors haven’t found a significant linear relationship between self-regulation and anxiety, they have identified negative relationship between self-efficacy and anxiety. Also they point out that students with high anxiety show less self-regulation and perseverance.

### The aim and importance of the study

Boekaerts and Corno (2005) list different instruments being used to assess self-regulation. The list includes self-report questionnaires, observations of overt behaviour, interview evidence, traces of mental events and processes, situational manipulations, recording student motivation strategies as they work, keeping diaries. They stress the insufficiency of using one instrument to observe students’ progress in self-regulation and they state that a combination of instruments should be used for such assessment purposes. Winne and Perry (2000) make a distinction between instruments that measure self-regulation as an aptitude (self-report questionnaires, structured interviews, and teacher judgments) and as an event (think aloud measures, error detection tasks, trace methodologies, and observations of performance).

The main purpose of the study was to develop and validate a self-report scale that could be used to evaluate self-regulated learning skills of university students (age 18 or above) in Turkey. As pointed out by Winne and Perry (2000: 542), self-report questionnaires are “… the most frequently used protocol for measuring SRL, perhaps because they are relatively easy to design, administer, and score…”. Self-report scale studies (e.g. Self-Regulatory Learning Inventory, SRLI of Lindner et al. 1996; Motivational Strategies for Learning Questionnaire, MSLQ of Pintrich and De Groot 1990; Pintrich et al. 1991; Self-Regulatory Learning Interview Schedule, SRLIS of Zimmerman and Martinez-Pons 1986; and Self-Regulated Learning Skill Inventory, SRLSI of Heo 1998) so far have focused on self-regulated learning skills of students at secondary and college level. Additionally, those scale studies have different frameworks; one scale consists of only cognitive and metacognitive learning strategies, while another one encompasses cognitive and metacognitive learning strategies and limited number of motivational dimensions involved in learning. Even there are some adaptation studies of original scales and questionnaires (Büyüköztürk et al. 2004) in Turkey. The authors thought of a need to handle more thoroughly the cognitive and metacognitive learning strategies together with the related motivational dimensions. Additionally the authors believed in the necessity of developing a scale that represented the self-regulatory skills of Turkish students, a scale custom to Turkish learners and their learning traditions. This is not an adaptation study, yet the framework was derived as a result of extensive review of related studies in literature. Since the authors thought that the construct of self-regulation could not be independent of motivation in learning or vice versa, they decided to form the scale of two sections or sub-scales: self-regulatory skills and motivation. This structure is in line with Pintrich, Zimmerman and Schunk’s notion of self-regulation.

According to the framework, there are two main sections. The self-regulated learning skills section covers 10 dimensions, whereas the motivation section covers 5. The dimensions in the self-regulated learning skills section are exactly the same as those in Zimmerman and Martinez-Pons’s Self-Regulated Learning Interview Schedule (SRLIS). To better reflect the construct of self-regulation, later those self-regulated learning skills dimensions were grouped under three main dimensions: before study, during study, after study. The sections and dimensions are summarized in Table 2.

**Table 2:** Self-regulation in learning scale: sections and dimensions

Self-regulated learning skills	Motivational factors
A. Before study	Self-efficacy
Goal setting and planning	Goal orientations
Environmental structuring	Task value
B. During study	Attributions for failure
Organization and transforming	Anxiety
Seeking information	
Rehearsing and memorizing	
Keeping records and self-monitoring	
Seeking peer, teacher or adult assistance	
Reviewing	
C. After study	
Self-evaluation	
Self-consequences	

## Methods

According to the details given above, the aim of this study was to primarily develop and validate a self-report scale on self-regulation for university students. For this particular purpose, the procedures and stages of implementation are given below separately.

### Overview of procedures

In the present study, several empirical studies were conducted to develop and validate the “Scale on Self-Regulation in Learning”. First, an original item pool pertaining to the content of self-regulation was generated and reduced. For this purpose, a pool of measurement items was collected from literature review and from those responses of students who answered open-ended questions about the characteristics of self-regulation and motivation in learning. Additionally, opinions of content area experts were called upon on the items in the pool and the number of items was reduced according to the suggestions of those experts.

Next, the initial form of the scale was administered to university students and the factor structure was determined by using exploratory and confirmatory factor analyses. Exploratory factor analysis, item-total correlations, and other reliability analyses (e.g. Cronbach’s alpha, item mean differences of top 27 % and bottom 27 %) were undertaken to assess the psychometric properties of the scale. Additionally, each dimension in the scale was evaluated through CFA to test their goodness of fit. According to Hair et al. (2010), the quality of fit depends heavily on model characteristics, including sample size and model complexity. They also state that multiple fit indices should be used to evaluate goodness-of-fit. In order to overcome conflicting conclusions resulting from sample size or model complexity, the following goodness of fit indices were used to test Model 1: the ratio of x^2^ and degrees of freedom (x^2^/df), goodness of fit index (GFI), normed fit index (NFI), root mean square residual (RMR), standardized RMR (SRMR), root mean square error of approximation (RMSEA), comparative fit index (CFI), incremental fit index (IFI), and adjusted goodness of fit index (AGFI). The perfect and acceptable values for goodness of fit indices suggested by Hair et al. (2010) and Schermelleh-Engel et al. (2003: 52) were taken basis in this study. SPSS 20.0 and LISREL 8.51 were utilized rigorously during all analyses.

### Participants

For the scale development process, data were collected from 1055 first to last year university students, whose ages ranged 18–22. A total of 872 students (83 %) responded. They rated each item using a 5-point scale with 1 = never, 2 = rarely, 3 = sometimes, 4 = usually, and 5 = always. The assistance of sixteen instructors was received during the administration. The participating students were all voluntary and were given clear instructions and enough time to complete the scale.

### Findings and results

The findings and results obtained during the scale development are explained in detail for each stage (Study 1 to 3) below under the title of development of the scale.

### The development of the scale

#### Study 1: generation and reduction of original item pool

To obtain a pool of measurement items that reflect the characteristics of self-regulating learners, an extensive literature review was carried out and also 38 university students were given open-ended questions about the 15 dimensions already described in the "Background" section. All the responses were collected, analyzed, and the items to be included in the scale were identified. Together with the items adapted and utilized from similar studies in literature (Self-Regulatory Learning Inventory, SRLI of Lindner et al. 1996; Motivational Strategies for Learning Questionnaire, MSLQ of Pintrich and De Groot 1990; Pintrich et al. 1991; Self-Regulatory Learning Interview Schedule, SRLIS of Zimmerman and Martinez-Pons 1986; and Self-Regulated Learning Skill Inventory, SRLSI of Heo 1998) and those derived from the responses students had given to the open-ended questions, the number of items in the scale rose up to 184. Six experts in the content areas of measurement and evaluation and curriculum and instruction reviewed and rated the items in terms of their relevance (1 = low, 2 = moderate, and 3 = high) to the identified content domains, their clarity and level of comprehensibility. Those statements with high ratings were chosen, which resulted in a smaller pool of 116 items (self-regulated learning skills section = 74 items, motivational dimensions section = 42 items). Later, this 116-item scale was given to 22 university students for a close inspection of the clarity and comprehensibility of its statements. Students submitted no problems with any of the items in the scale, so this process resulted in 116 items being developed for scale purification process (exploratory and confirmatory analyses). The total score of the scale ranged from 116 to 580.

#### Study 2: exploratory and confirmatory factor analyses for construct validity of the scale

Basic statistics were calculated according to results of initial scale administration. The fact that the score variance was high (2523.07), the scores had a wide range (309) and the values of mean, median and mode were close to each other showed that the scores obtained from the administration had a normal distribution. The Kaiser–Meyer–Olkin (KMO) measure of sampling adequacy index of 0.90 and the Bartlett’s test of sphericity index of 39,845.20 (p < 0.01) provided evidence that the data were suitable for principal components analysis (or factor analysis). Also the Cronbach’s Alpha coefficient for the initial scale was calculated as 0.95 and this suggested that the scale had high internal-consistency.

The analyses included a combination of reliability analysis with a particular attention being given to corrected item-to-total correlations and exploratory factor analysis (EFA). Item-to-total correlations were examined and those calculated at less than 0.40 were identified for possible deletion. This was followed by an examination of items that did not load strongly (i.e. loadings of <0.40) and had high cross-loadings from factor analysis results. As a result of this analysis:

- In the “Before Study” main dimension;
  - The “Goal setting and planning” dimension was split into two and relabeled as “Arrangement of study time” and “Planning”,
  - Four items were deleted due to weak loadings and high cross loadings.
- In the “During Study” main dimension;
  - The “Seeking information” dimension was split into two and relabeled as “Seeking appropriate information” and “Seeking easily accessible information”,
  - Likewise, the “Keeping records and self-monitoring” dimension was split into two and relabeled as “Self-monitoring” and “Keeping records of learning”,
  - The “Reviewing” dimension was removed from the scale, because of no strong loadings at all,
  - Finally, thirteen items were removed due to weak loadings and high cross loadings.
- In the “After Study” main dimension; “The “Self-consequences” dimension was split into two and relabeled as “Self-consequences after success” and “Self-consequences after failure”.
- In the “Motivation” section;
  - The dimensions perfectly fit the data, so the labels remained the same,
  - Just fourteen items were deleted due to weak loadings and high cross loadings.

An 18-dimension solution resulted from principal components factor analysis using varimax and oblique rotations. The factor loadings ranged from 0.43 to 0.91. Furthermore, Cronbach’s alpha was computed at 0.93, which indicated strong internal consistency of the scale. This 85-item-version of the scale was named as Model 1.

**Fig. 1:**
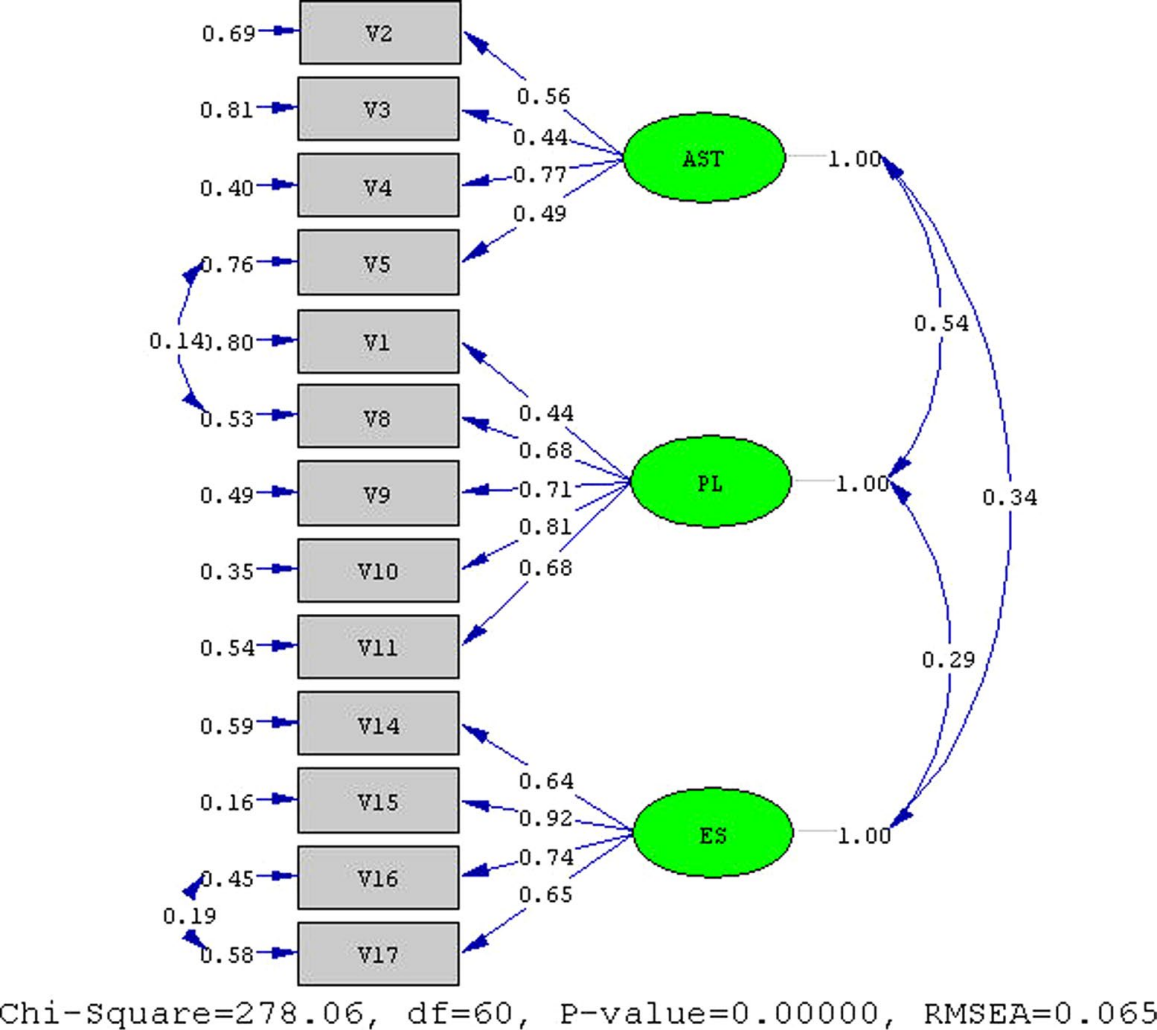
CFA result of before study main dimension. *AST* arrangement of study time, *PL* planning, *ES* environmental structuring

**Fig. 2:**
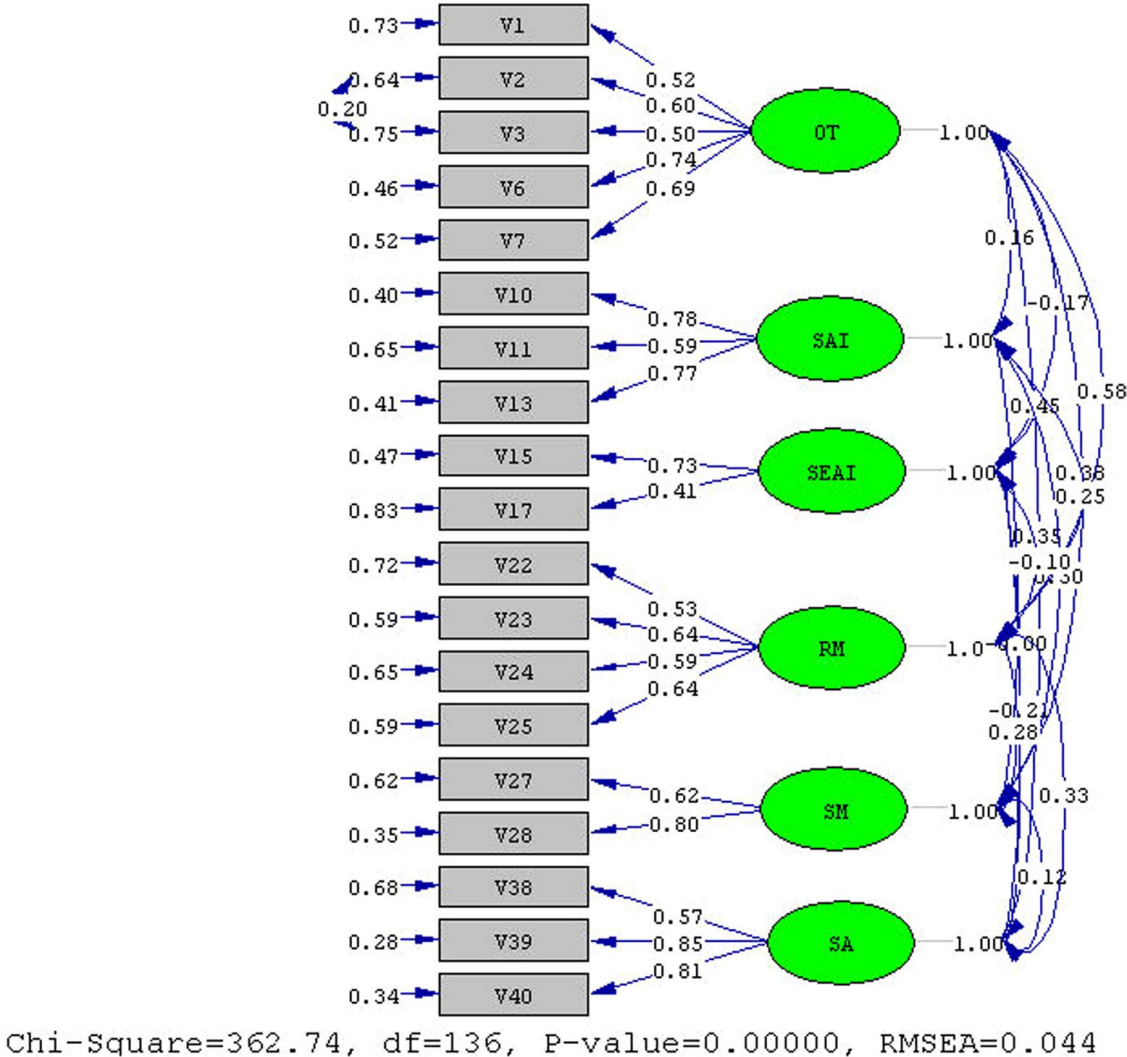
CFA result of during study main dimension. *OT* organization and transforming, *SAI* seeking appropriate information, *SEAI* seeking easily accessed information, *RM* rehearsing and memorizing, *SM* self-monitoring, *SA* seeking assistance

**Fig. 3:**
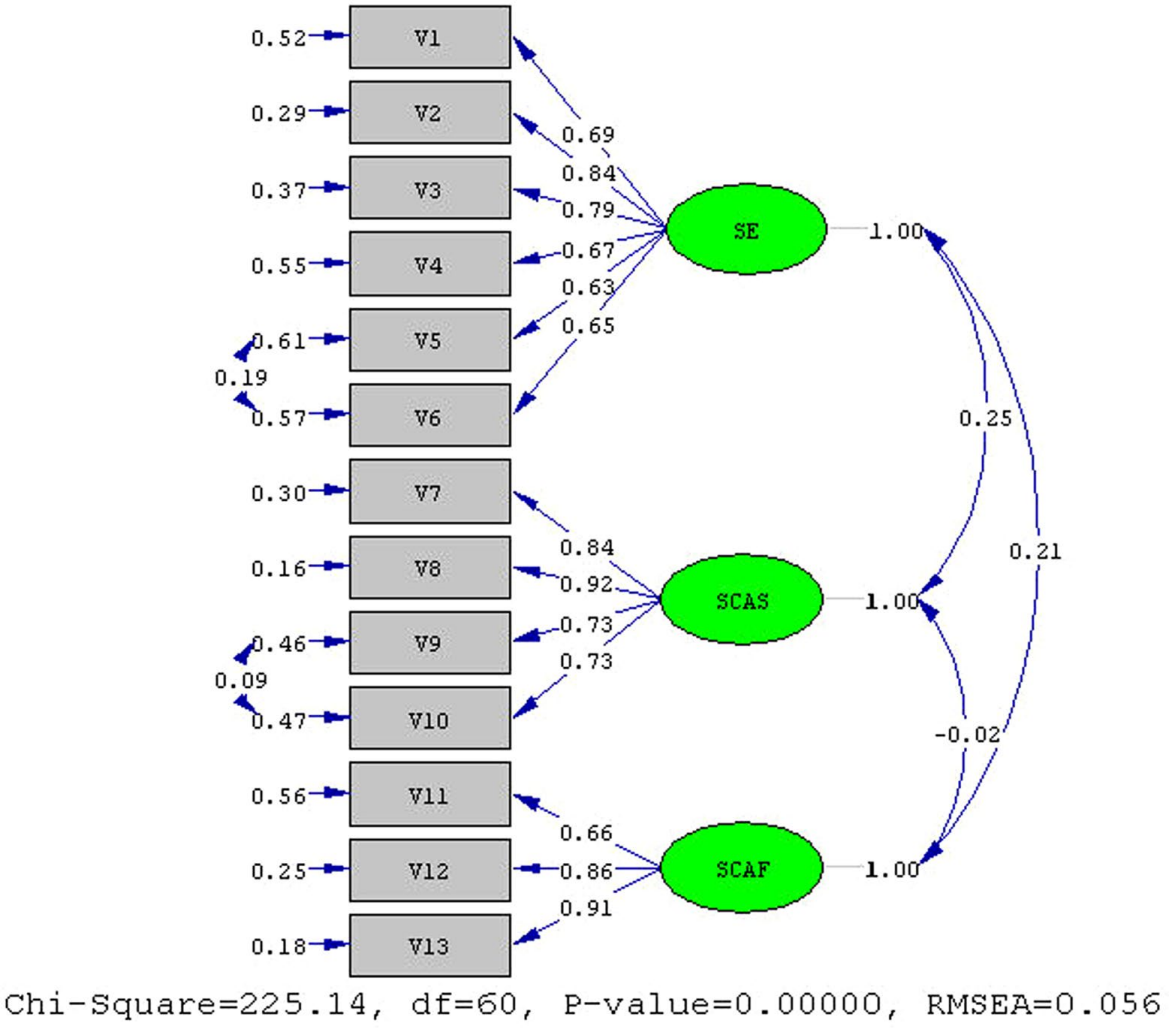
CFA result of after study main dimension. *SE* self-evaluation, *SCAS* self-consequences after success, *SCAF* self-consequences after failure

**Fig. 4:**
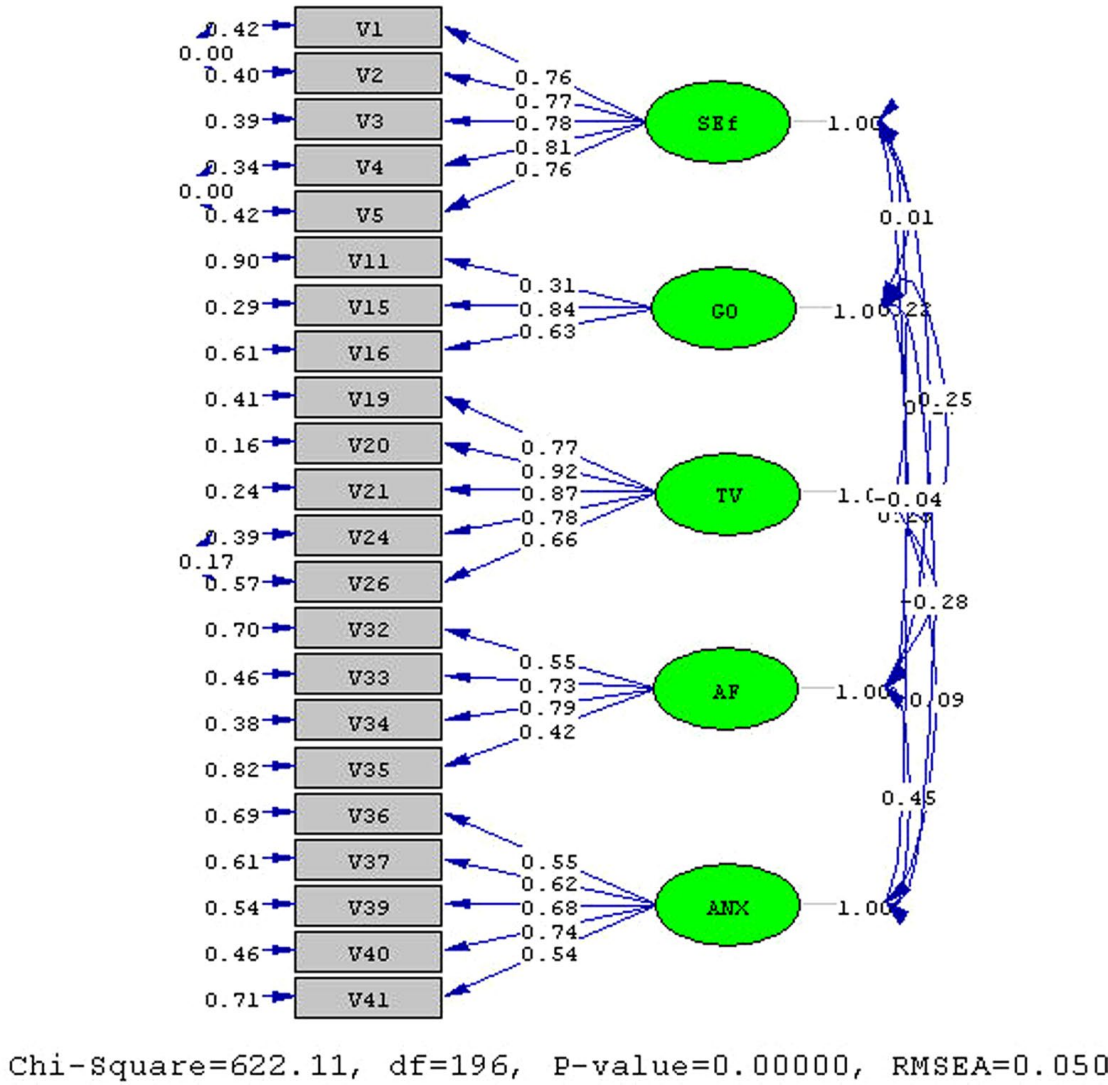
CFA result of motivational dimensions section. *SEf* self-efficacy, *GO* goal orientations, *TV* task value, *AF* attributions for failure, *ANX* anxiety

Later, the 85-item “Scale on Self-Regulation in Learning” (Model 1) was evaluated through confirmatory factor analysis. LISREL 8.51 (Karl Jöreskog and Dag Sörbom 2001) was used for that purpose. When the goodness-of-fit indices for each dimension were analyzed, poor fit statistics was found with “during study” main dimension and “motivational dimensions” section of the scale. After examination of the modification indices of the CFA analysis for each dimension; the “keeping records of learning” dimension was removed together with twelve items from the “during study” main dimension and six items were deleted from the “motivational dimensions” section. The CFA was re-examined on the remaining 17 dimensions and 67 items, resulting in sound fitness of the model (Model 2). The results of CFA are presented in Figs. 1, 2, 3, 4.

Table 3 summarizes goodness-of-fit indices for Model 1 and Model 2. Although there are no well-established guidelines for what minimal conditions constitute an adequate fit, some rules of thumb exist. Further analyses of the indices suggested that they were at least within the acceptable criteria range, as recommended by Schermelleh-Engel et al. (2003) and Hair et al. (2010). These findings suggested that the model (Model 2) fit the sample data, or in other words, matched the observed data.

**Table 3:** Comparison of Model 1 and Model 2 confirmatory factor analysis goodness-of-fit indices

Dimensions	Models	Goodness-of-fit Statistics								
		*x* ^*2*^/*df*	GFI	AGFI	SRMR	RMR	CFI	RMSEA	NFI	IFI
Before study	1	4.6	0.95	0.93	0.04	0.10	0.94	0.07	0.93	0.94
	2	No change, the same with Model 1								
During study	1	4.3	0.88	0.86	0.07	0.10	0.83	0.06	0.79	0.83
	2	2.7	0.96	0.94	0.04	0.06	0.94	0.04	0.91	0.94
After study	1	3.8	0.96	0.94	0.05	0.08	0.97	0.06	0.96	0.97
	2	No change, the same with Model 1								
Motivation	1	7	0.84	0.80	007	0.10	0.85	0.08	0.82	0.85
	2	3.2	0.94	0.92	0.05	0.07	0.95	0.05	0.93	0.95

Exploratory factor analysis was conducted again with the 17-dimension, 67-item Model 2. The factor loadings ranged from 0.47 to 0.91 (refer to Table 4 for the factor loadings and variance each dimension explains).

**Table 4:** Factor loadings and total variance explained by each dimension in Model 2

Factor	Number of items	Factor loadings	Total variance explained
*Before study*			
Environmental structuring	4	0.69–0.83	3.95
Planning	5	0.57–0.77	3.87
Arrangement of study time	4	0.60–0.76	2.40
*During study*			
Organization and transforming	5	0.56–0.83	3.78
Seeking appropriate information	3	0.72–0.73	3.13
Seeking peer, teacher or adult assistance	3	0.74–0.86	3.33
Seeking easily accessible information	2	0.65–0.89	2.33
Self-monitoring	2	0.80–0.84	2.48
Rehearsing and memorizing	4	0.68–0.73	3.31
*After study*			
Self-evaluation	6	0.72–0.84	5.99
Self-consequences after success	4	0.84–0.90	4.72
Self-consequences after failure	3	0.79–0.91	3.86
*Motivation*			
Task value	5	0.76–0.89	5.84
Self-efficacy	5	0.79–0.84	5.46
Anxiety	5	0.62–0.79	4.17
Attributions for failure	4	0.59–0.78	3.39
Goal orientations	3	0.47–0.83	2.48

Additionally, the analysis of the correlations between the sections and main dimensions in the scale (Table 5) showed that they correlated well with each other in expected directions.

**Table 5:** Correlation coefficients between section and main dimension scores in the Scale. ** Correlation is significant at the 0.01 level (2-tailed)

	Scale total	Learning skills	Motivation	Before study	During study
Learning skills	0.942**				
Motivation	0.757**	0.494**			
Before study	0.807**	0.846**	0.443**		
During study	0.782**	0.851**	0.369**	0.590**	
After study	0.796**	0.832**	0.444**	0.584**	0.527**

#### Study 3: reliability analyses

The reliability for this scale, as calculated via Cronbach’s alpha, was confirmed at 0.91 (Before Study = 0.78, During Study = 0.77, After Study = 0.82, and Motivation = 0.81 were found separately). As part of item analysis, the item-total test correlations were calculated and by using unrelated *t* test analysis the relationship between item means of students at top quartile (27 %) and bottom quartile (27 %) were examined. The results of these applications, which indicate the scale’s internal consistency and item discrimination (Büyüköztürk 2002: 171–172), are shown in Table 6.

**Table 6:** Item analysis results of the final scale

General dimensions	Item no.	Item-total correlation^1^	t (Bottom 27 %–Top 27 %)^2^	General dimensions	Item no.	Item-total correlation^1^	t (Bottom 27 %–Top 27 %)^2^
Before Study	1	0.33	13.36*	After study	33	0.51	20.34*
	2	0.37	16.99*		34	0.61	25.34*
	3	0.25	10.59*		35	0.58	23.45*
	4	0.48	20.85*		36	0.49	20.67*
	5	0.36	14.63*		37	0.49	18.51*
	6	0.52	21.29*		38	0.53	21.63*
	7	0.44	17.58*		39	0.48	19.57*
	8	0.55	23.39*		40	0.48	18.76*
	9	0.42	16.15*		41	0.47	17.61*
	10	0.37	13.98*		42	0.38	13.68*
	11	0.47	15.87*		43	0.36	12.61*
	12	0.42	14.43*		44	0.25	9.91*
	13	0.37	12.92*		45	0.29	11.22*
During study	14	0.40	13.97*	Motivation	46	0.36	13.03*
	15	0.44	15.73*		47	0.39	13.26*
	16	0.42	16.44*		48	0.43	14.40*
	17	0.44	16.53*		49	0.45	15.16*
	18	0.46	16.52*		50	0.46	15.76*
	19	0.33	12.53*		51	0.26	10.31*
	20	0.23	9.03*		52	0.29	11.21*
	21	0.37	13.02*		53	0.32	12.84*
	22	0.33	12.53*		54	0.49	19.36*
	23	0.32	12.84*		55	0.57	21.18*
	24	0.38	13.92*		56	0.52	20.34*
	25	0.41	15.98*		57	0.56	21.21*
	26	0.41	16.28*		58	0.46	16.36*
	27	0.42	15.27*		59	0.34	12.97*
	28	0.29	11.21*		60	0.40	15.04*
	29	0.32	12.84*		61	0.47	18.18*
	30	0.21	7.86*		62	0.26	10.31*
	31	0.36	12.80*		63	0.29	10.48*
	32	0.36	12.31*		64	0.34	12.75*
^1^n = 872	^2^n_1_ = n_2_ = 235	*p < 0.01			65	0.37	11.82*
					66	0.28	10.68*
					67	0.29	10.78*

## Discussion

After the thorough process of scale development for the “Scale on Self-Regulation in Learning”, the results indicated that self-regulation was a multidimensional construct that differentiated individuals. The existence of the two sections in this scale, as theorized and constructed from the findings in literature, was supported by the exploratory and confirmatory factor analyses.

Using the entire sample, the confirmatory factor analysis on the 67-item version showed acceptable fit for the 17-dimension model (refer to Table 3 for the goodness-of-fit indices). The loadings ranged from 0.47 to 0.91 (refer to Table 4 for the factor loadings and variance each dimension explains). Furthermore, Cronbach’s Alpha coefficient for the whole scale was computed at 0.91 (0.78, 0.77, 0.82, and 0.81 were alphas found for Before Study, During Study, After Study, and Motivation respectively), which indicated strong internal consistency of the scale.

As part of item analysis, the item-total test correlations were calculated and by using unrelated/uncorrelated *t* test analysis the relationship between item means of students at top quartile (27 %) and bottom quartile (27 %) was examined. The results of these again indicated that the scale has high internal consistency and the scale items discriminate well among students.

The Self-Regulatory Learning Inventory (SRLI) of Lindner et al. (1996) includes four subscales: executive processing, cognitive processing, motivation, and environmental control and utilization. The internal reliability of this 80-item scale ranges from 0.78 to 0.93. The Motivational Strategies for Learning Questionnaire (MSLQ) of Pintrich and De Groot (1990) includes 81 items on learner motivation, cognitive strategy use, metacognitive strategy use, and management effort and has reliability ranging from 0.74 to 0.89. The Self-Regulatory Learning Interview Schedule (SRLIS) of Zimmerman and Martinez-Pons (1986) identifies 14 classes of self-regulated behavior that can occur in six learning contexts. It asks learners to indicate how they participate in class, how they study and complete their assignments. The Self-Regulated Learning Skill Inventory (SRLSI) of Heo (1998) encompasses five categories with 29 items: motivation and self-efficacy, cognitive strategy, metacognitive strategy, environmental utilization, and epistemological beliefs and has the reliability of 0.76. In comparison with these highly acclaimed scales on SRL, the present scale with its focus both on students’ motivational orientations and various learning skills/strategies includes 17 dimensions with 67 items and has the reliability of 0.91. The internal reliability of the main dimensions of the scale (before study, during study, after study, and motivation) ranges from 0.78 to 0.82. In light of such a fact, the authors believe that the present scale could be used reliably to measure levels of student self-regulation at university level, and other age and/or grade levels in Turkey. It is expected that replication of the scale with more students from different levels will increase its reliability in time.

The results provided even more conclusive evidence for the fact that self-regulated learning rests both on learning skills/strategies and motivational factors. As depicted in the introduction part, self-regulation cannot be thought regardless of such motivational components like self-efficacy, task value, or goal orientations. One’s learning skills/strategies and his/her motivation are in constant interaction with each other. For example; frequent use of self-regulated skills/strategies brings success and eventually builds up self-efficacy, in return high self-efficacy leads to more resort to those learning skills/strategies that bring success. As also stated by many scholars (McCombs 2001; Pintrich and De Groot 1990; Zimmerman 1990), the findings of this study reiterates the fact that self-regulation or self-regulated learning should be comprised of not only learning skills/strategies, but also motivational factors as well.

It is also worth mentioning that this collection does not include all possible regulation of cognitive and motivation strategies, it is a cross-sectional representation of the ways which university students in Turkey use to manage their cognitive and motivational processes. Another concern with the scale presented here is the relative lack of empirical data specifically examining its validity with regard to particular individual differences like gender, age level, and socioeconomic status. Additional research examining the psychometric properties of this scale within diverse populations is needed to provide additional evidence regarding this aspect of validity.

The authors main intent was to develop a self-report scale that could be used to measure the self-regulatory learning levels of university students in Turkey. Turner (1995) points to the relative ease of designing, administering, and scoring self-report questionnaires and adds that they provide (a) information about learners’ memories and interpretations of their actions and (b) their explanations of cognitive and metacognitive processes researchers cannot observe (in Winne and Perry 2000: 542). Keeping such reviews in mind, the authors thought that such a self-report measure would be helpful to researchers and/or teachers dealing with large groups of students. However, as proposed by Boekaerts and Corno (2005), one instrument would not be sufficient enough to fully observe students’ self-regulation in progress. Supporting findings of instruments that measure self-regulation as an aptitude (like self-report questionnaires) with those that measure self-regulation as an event (like think alouds and observations) would definitely provide the researchers or teachers with better pictures of their students’ progress in self-regulation.

## Conclusions and recommendations

In this study, the development and preliminary validation of a new Turkish measure of self-regulation in learning for university students was described. After all, a parsimonious, 17-dimensional, 67-item scale, demonstrating reliability and validity, resulted from this development process. The resulting scale consisted of two sub-scales or sections: self-regulated learning skills and motivational factors. Self-regulated learning skills (or cognitive factors) were grouped under three main dimensions, namely before study (environmental structuring, planning, and arrangement of study time), during study (organizing and transforming, seeking appropriate information, seeking easily accessible information, seeking peer, teacher or adult assistance, self-monitoring, and rehearsing and memorizing), and after study (self-evaluation, self-consequences after success, and self-consequences after failure). The motivational factors included task value, self-efficacy, anxiety, attributions for failure, and goal orientations. The scale is in Turkish. The total number of items together with English translation of sample items for each dimension is given in the “Appendix”.

The authors believe that this self-report measure can be used to evaluate self-regulated learning skills of university students in Turkey and if validated could be used with other age groups as well. The different sections or sub-scales and main dimensions on SSRL can be used together or singly. The sections and main dimensions are designed to be modular and can be used to fit the needs of the researcher or instructor. The results of the scale can be used for feedback or profiling purposes. For example; students in a class could compare their scores they get from the whole scale, sections or dimensions of the scale to see in which areas they are good or they need improvement or assistance. Likewise, average scores could be used to set the profiles of students individually or in groups. The instrument is designed to be given in class and takes approximately 20–25 min to administer. The total set of items may be available for bona fide research purposes, if required from the corresponding author.

This is a self-report instrument. It is essential to support the results of the applications of this scale with other measures of self-regulation (observations or interviews) if deeper understanding of student progress with respect to self-regulatory processes is intended. Notwithstanding insufficiencies of such self-report questionnaires, this measure is easy to administer and score when used with large bodies of students.

The aim of this study was to develop a scale on self-regulation, so individual differences like gender, age level, and socioeconomic status and the possible influence of powerful learning environments were not taken into consideration. The authors’ intention is to handle these factors in their future research to provide more evidence for the validity of this scale.

## Appendix

See Table 7.

**Table 7:** Total item numbers and samples from “Scale on Self-Regulation in Learning-SSRL”

Dimensions	Number of items	Sample items
*Before study*		
Environmental structuring	4	I usually study where I can focus
Planning	5	I write my weekly to-do-list in my notebook
Arrangement of study time	4	I do my homework when I know our teacher will control them
*During study*		
Organization and transforming	5	I find the key points in the text and draw connections between them.
Seeking appropriate information	3	I read the sources I find after class
Seeking peer, teacher or adult assistance	3	When I don’t understand, I seek the assistance of a peer or an adult.
Seeking easily accessible information	2	I try to find the easiest way of doing my homework
Self-monitoring	2	While reading a book or reviewing my notes, I sometimes stop and ask myself, “Do I understand the point here?”
Rehearsing and memorizing	4	I teach the topic I study to another person
*After study*		
Self-evaluation	6	Generally I don’t revise a homework I have finished
Self-consequences after success	4	I promise to award myself after I get a good grade from an exam or homework
Self-consequences after failure	3	Failures make me sad, but I don’t do anything to change them
*Motivation*		
Task value	5	I believe we’ll use the things we learn in class in the future
Self-efficacy	5	I think I’ll succeed in the courses
Anxiety	5	I get so excited in exams that I forget everything
Attributions for failure	4	Extreme load of homework and exams makes me fail
Goal orientations	3	The most satisfactory thing for now is getting a high grade
